# The Impact of the ‘Mis-Peptidome’ on HLA Class I-Mediated Diseases: Contribution of ERAP1 and ERAP2 and Effects on the Immune Response

**DOI:** 10.3390/ijms21249608

**Published:** 2020-12-17

**Authors:** Valentina Tedeschi, Giorgia Paldino, Fabiana Paladini, Benedetta Mattorre, Loretta Tuosto, Rosa Sorrentino, Maria Teresa Fiorillo

**Affiliations:** 1 Department of Biology and Biotechnology ‘Charles Darwin’, Sapienza University, 00185 Rome, Italy; giorgia.paldino@uniroma1.it (G.P.); fabiana.paladini@uniroma1.it (F.P.); benedetta.mattorre@uniroma1.it (B.M.); loretta.tuosto@uniroma1.it (L.T.); rosa.sorrentino@uniroma1.it (R.S.); mariateresa.fiorillo@uniroma1.it (M.T.F.); 2 Laboratory Affiliated to Istituto Pasteur Italia-Fondazione Cenci Bolognetti, Sapienza University, 00185 Rome, Italy

**Keywords:** HLA class I molecules, ERAP1 and ERAP2 ER aminopeptidases, immunopeptidome, autoimmune/autoinflammatory disorders, antigen presentation, CD8^+^ T cells

## Abstract

The strong association with the Major Histocompatibility Complex (MHC) class I genes represents a shared trait for a group of autoimmune/autoinflammatory disorders having in common immunopathogenetic basis as well as clinical features. Accordingly, the main risk factors for Ankylosing Spondylitis (AS), prototype of the Spondyloarthropathies (SpA), the Behçet’s disease (BD), the Psoriasis (Ps) and the Birdshot Chorioretinopathy (BSCR) are HLA-B*27, HLA-B*51, HLA-C*06:02 and HLA-A*29:02, respectively. Despite the strength of the association, the HLA pathogenetic role in these diseases is far from being thoroughly understood. Furthermore, Genome-Wide Association Studies (GWAS) have highlighted other important susceptibility factors such as Endoplasmic Reticulum Aminopeptidase (ERAP) 1 and, less frequently, ERAP2 that refine the peptidome presented by HLA class I molecules to CD8^+^ T cells. Mass spectrometry analysis provided considerable knowledge of HLA-B*27, HLA-B*51, HLA-C*06:02 and HLA-A*29:02 immunopeptidome. However, the combined effect of several ERAP1 and ERAP2 allelic variants could generate an altered pool of peptides accounting for the “mis-immunopeptidome” that ranges from suboptimal to pathogenetic/harmful peptides able to induce non-canonical or autoreactive CD8^+^ T responses, activation of NK cells and/or garbling the classical functions of the HLA class I molecules. This review will focus on this class of epitopes as possible elicitors of atypical/harmful immune responses which can contribute to the pathogenesis of chronic inflammatory diseases.

## 1. Introduction

The MHC (Major Histocompatibility Complex) class I and II molecules, known as HLA (Human Leucocyte Antigen) in humans, are essential for promoting specific immunity; in particular, HLA class I molecules elicit CD8^+^ T cell responses directed against epitopes, usually nine residues in length, derived from endogenously synthesized microbial or cellular proteins [1]. These peptides, upon the N-terminal refinement by the Endoplasmic Reticulum Aminopeptidases (ERAP) 1 and 2, are accommodated into the groove of the HLA class I molecules through the so-called “anchor” residues that are embedded into specific pockets [2]. In particular, residues at position 2 (P2) and at the carboxy-terminal (PΩ) are pivotal for the correct placement of the peptides through connections with the B and F pocket, respectively [3,4]. The binding motif is generally conserved for each HLA allele. However, some circumstances, such as an inflammatory environment or the use of specific drugs concomitantly with particular ERAP1 and ERAP2 haplotypes, could allow the binding cleft to assume conformations that can become permissive for unconventional peptides. In this context, it is of interest the case of drug-hypersensitivity induced by abacavir in HLA-B*57:01 positive subjects or by carbamazepine in HLA-B*15:02 carriers [5]. In particular, the non-covalent interaction of the drug with the F pocket of HLA-B*57:01 dramatically alters the self-epitope repertoire displayed by the HLA molecule creating foreign complexes, which induce robust T cell responses in the HLA-B*57:01 carriers taking abacavir [6,7,8]. Moreover, several viruses (i.e., HIV, CMV, EBV) could contribute to the alteration of the peptide repertoire establishing their strategies to escape immune surveillance [9].

Ankylosing Spondylitis (AS), Psoriasis (Ps), Birdshot Chorioretinopathy (BSCR) and Behçet’s disease (BD) are referred as “MHC-I-opathies” as they share an association with HLA-class I genes, in particular HLA-B*27, HLA-C*06:02, HLA-A*29:02 and HLA-B*51, respectively [10]. Such diseases also share ERAP1 and, in some cases, ERAP2 as susceptibility factors and display as overlapping common targets, tissues undergoing either mechanical (enthesis and bone) or environmental stress such as skin, oral mucosa, gut and eye. In spite of different manifestations, a common basis of these diseases at the crossroads between the innate and adaptive immune system, which culminates in the typical chronic inflammation, has been suggested [10].

Over time, several case-control association analyses and Genome-Wide Association Studies (GWAS) have robustly shown associations of Single Nucleotide Polymorphisms (SNPs) in ERAP1 and/or ERAP2 genes or even of entire haplotypes with the above-mentioned diseases [11,12,13,14,15,16,17,18]. Functional effects of this ERAP1 and 2 variance has also been investigated but little is known about the molecular mechanisms in the critical cells. Moreover, few genetic studies have been focused on ERAP gene promoters and on the mechanisms regulating gene expression [19]. In the case of ERAP1, ten haplotypes (Hap1 to Hap10), derived from a combination of multiple non-synonymous SNPs, account for over 99% of the natural ERAP1 variants; however, the association of these haplotypes with each disease is quite different [20]. The striking association with HLA class I molecules and the involvement of ERAPs would point out a central role for CD8^+^ T cells or even Natural Killer (NK) cells in tissue-specific damage. Accordingly, an altered antigen presentation could be one of the possible mechanisms behind the autoimmune injury caused by some haplotypes of ERAP1 and ERAP2, which are pivotal in the processing of HLA class I epitopes.

In this review, we will focus on the so-called “mis-peptidome” by discussing a potential harmful role of the suboptimal and self-immunopeptidome in the “MHC-I-opathies”, the influence of ERAP1 and ERAP2 allelic variants and the implication in the onset of AS and related SpA, BD, Ps and BSCR.

## 2. HLA-B*27 and Spondyloarthropathies: Beyond the ‘Classical’ Peptidome

The HLA-B*27 is one of the most investigated HLA class I molecule that came to the attention during the early 1970s for its association with AS and other related inflammatory disorders collectively known as seronegative Spondyloarthropathies (SpA), which comprise Psoriatic Arthritis (PsA), Reactive Arthritis (ReA), Anterior Uveitis-associated Arthritis and the Arthritis linked to Inflammatory Bowel Disease (IBD). However, an univocal explanation for this remarkable linking is still lacking [21,22]. Several scenarios take into account not only the canonical function of HLA-B*27 as peptide presenting molecule to cytotoxic CD8^+^ T lymphocytes (CTLs) or ligand for NK receptors, but also some “aberrant” features such as misfolding and homodimerization [23]. On the other hand, the HLA-B*27 has been described as protective factor against several viral infections (HIV, HCV, EBV and influenza virus), probably due to a better performance of the virus specific HLA-B*27-restricted CD8^+^ T cells [17,24]. Ideally, the common ground of these two aspects could be the peptidome. In fact, the quality and the quantity of peptides available for the binding to the HLA-B*27 molecules could affect their stability and function. Investigating the immunopeptidome is therefore useful to design more specific therapies.

HLA-B*27 is highly polymorphic with more than 200 subtypes [25] identified so far, but not all associated with AS [26]. Of note, most of the variance is located in the binding groove cleft, substantiating the relevance of the peptide repertoire. Despite the attempts to identify a specific peptidome displayed by the AS-associated molecules (HLA-B*27:05, -B*27:02, -B*27:04 and -B*27:07) versus the non-AS-associated ones (HLA-B*27:06 and -B*27:09) no definitive answer has emerged as yet [27,28]. However, it is still worth pursuing this goal focusing on a pair of alleles that show a different association with AS such as HLA-B*27:05 and HLA-B*27:09, differing for a single amino acid (Asp116His), or B*27:04 and B*27:06 differing for two amino acids [29,30].

The HLA-B*27 peptidome consists mainly of 9-mers and 10-mers [31] with Arg at P2 and basic, aliphatic or aromatic residues at C-terminus [18]. Although a hallmark of the B27 peptidome is an Arg as main anchor at P2, recent studies have introduced Gln and Lys as alternative P2 residues, albeit much lower represented compared to Arg [32,33]. In this context, Lorente and colleagues have shown that these two amino acids are enriched at P2 in the HLA-B*27:05 ligandome when ERAP2 is absent [34]. The authors also found an increase of basic residues at N-terminal with a concurrent reduction of hydrophobic amino acids at P3, P7 and P9 [34].

Before migrating to the cell surface, the HLA-B*27 molecules load the peptide cargo in the Endoplasmic Reticulum (ER). Interestingly, the tendency of HLA-B*27 molecules to fold slowly and to be retained in the ER in a peptide-receptive state could give to suboptimal peptides the chance to accommodate into the groove at the expense of classical B27 epitopes [35]. The peptide loading efficiency of HLA-B*27 molecules is therefore the result of their receptive state and is strongly influenced by qualitative and quantitative fluctuation of ERAP1 and 2. Notably, there is an epistatic gene-gene interaction between HLA-B*27 and ERAP1, since the association of ERAP1 with AS occurs only in HLA-B*27 positive patients, whereas the association of ERAP2 is independent of HLA-B*27, suggesting for ERAP2 a role distinct from antigen processing [11,12,36,37]. Now we know that the combination of highly active ERAP1 variants (Hap1 to Hap3) and the presence of ERAP2 could promote AS, whereas the opposite seems to be protective [20]. It is not clear whether this could be due to a “disruption” of canonical B27 peptides with consequent generation of new potentially pathogenetic epitopes [38]. In particular, the co-presence of ERAP2 with highly active ERAP1 variants (Hap1 and Hap2) favored an increase of 9-mers with a reduction of basic P1 residues [39]. Moreover, studies from human cells and transgenic rats showed that the depletion of ERAP1 induced the switch of the B27 peptidome towards longer peptides, C-terminally extended, and an alteration on the frequency of P1 residues [40,41]. Notably, longer peptides have been already described as intermediates or accidentally bound peptides with a lower frequency of the Arg in P2 [42]. In addition, the silencing or the inhibition of ERAP1 seemed to promote the reduction of FHC (Free Heavy Chain) homodimers on the surface of C1R and HeLa stably expressing HLA-B*27 as well as on monocytes from AS patients [43], suggesting that lowering the activity of ERAP1 as in the case of protective variants could preserve the stability of HLA-B*27 molecules providing an optimal ligandome.

The nature of the peptidome efficiently bound and presented by HLA-B*27 is highly influenced by the stability of the molecule itself; several studies have described a higher flexibility of HLA-B*27:05, compared to B*27:09, suggesting an intrinsic propensity of this subtype to adopt unconventional conformations, especially in the proximity of the binding groove [44,45,46]. Moreover, a recent work by Loll’s research group has suggested that HLA-B*27:05, but not B*27:09, undergoes metal ion-induced conformational alterations that, in turn, could influence the capability of the AS-associated allele to bind suboptimal peptides [47].

Our group has recently described a polymorphism, SNP rs75862629, located in the intergenic region between ERAP1 and ERAP2 that can affect the expression of both genes and, consequently, of HLA-B*27 molecules [19,48]. In particular, we showed that in the Sardinian population, the G variant, implicating high ERAP1 and low ERAP2 levels, induces a reduction in the surface expression of HLA-B*27:09, but not of HLA-B*27:05 [48]. In this regard, it is known that increased levels of HLA-B*27 expression also promote AS susceptibility [49]. In principle, the “disruption” of the conventional B27 peptidome due to the over-trimming by ERAP1 could cause an increase in suboptimal peptides that are not suitable for the B*27:09 binding but good enough for maintaining B*27:05 stability on the cell surface. As we recently reported, the higher flexibility of the HLA-B*27:05 binding groove and its low dependence on a classical B27 peptidome allow HLA-B*27:05 to present a HLA-B*07 restricted viral peptide (pEBNA3A-RPPIFIRRL) lacking the B27 consensus motif (Arg2) and nevertheless promoting the activation of specific CD8^+^ T cells. This response is not evoked by the HLA-B*27:09, remarking the lower stringency of the peptide repertoire bound by HLA-B*27:05 [50,51]. At present, we are investigating a possible correlation between this particular CD8^+^ T response in the context of HLA-B*27:05 and a specific haplotype of ERAP1 and ERAP2 (unpublished data). In this context, Rowntree et al. reported that several peptides derived from viruses causing chronic infections (EBV, CMV and HIV-1), usually displayed by the HLA-A*02, HLA-B*07 or HLA-B*57, could induce alloreactive CD8^+^ T cell responses towards HLA-B*27 [52]. Consistently, we also showed that the HLA-B*27 molecules can present viral epitopes introduced by chimeric proteins driven by TAT of HIV through a proteasome- and TAP-independent pathway, pointing out again the high flexibility of the HLA-B*27 molecules [53].

Another important aspect is the subclinical gut inflammation occurring in up to 70% of patients with AS as a possible consequence of microbial dysbiosis [54]. The alteration of the gut microbiota could be, at least in part, related to the activation of autoreactive CD8^+^ T cells towards microbial peptides displayed by HLA-B*27 [55]. Accordingly, the permissive binding of HLA-B*27:05 could enlarge the spectrum of the candidate epitopes.

The complexity of B27 peptidome, especially of B*27:05, is far from an unambiguous definition; matching mass spectrometry analyses with functional studies, could shed light on its double role as a protective factor in viral infections and as a predisposing molecule in the autoimmune diseases.

## 3. HLA-B*51 and Behçet’s Disease: Discovering the Multiple Facets of the Peptidome

Behçet’s disease (BD) is an inflammatory autoimmune disorder that mainly causes recurrent vasculitis and oral and genital ulcers. The classical clinical picture can extend to other sites including different organs and tissues such as eye, skin and bowel. In addition, neuronal symptoms may also arise [56]. The basis of BD is not fully understood but a strong correlation with the presence of the HLA-B*51 allele has been evidenced [57]. Studies on different ethnic groups highlighted the strong association of BD with the HLA-B*51:01 subtype [58,59]. So far, several theories have been proposed to define the mechanisms linking HLA-B*51 to BD and, similarly to the case of HLA-B*27 and AS, they reflect intrinsic properties of the molecule (i.e., being a “slow-folding” MHC molecule during peptide loading) and extrinsic features implying the presentation of self-peptides to autoreactive CD8^+^ T cells or NK cells [60,61]. A role for T cells in BD is sustained by the observations that lymphocyte-targeting drugs (such as azathioprine) are therapeutically beneficial [62]. Therefore, the idea of an altered spectrum of peptides displayed by HLA-B*51, also supported by the contribution of ERAP1 as a BD genetic risk, is quite appealing, although not conclusive.

Interestingly, HLA-B*51 differs from HLA-B*52, which is not associated with BD, for only two residues at position 63 (Asp63Glu) and 67 (Phe67Ser) located in the B pocket of the groove and mainly involved in the interaction with the P2 peptide anchor residue [63,64]. Similarly to HLA-B*27, HLA-B*51 may present unconventional endogenous peptides, potentially pathogenetic [65]. The majority of peptides bound to HLA-B*51 are 9-mers possessing Pro or Ala at P2 with Ile, Val and Leu as C-terminal residues. In particular, Pro seems to be a stronger anchor residue than Ala [66,67]. Noteworthy, beside Pro2 or Ala2 peptides, representing two distinct high affinity HLA-B*51 sub-peptidomes, the proteomic studies have highlighted non-conventional peptidomes non-Pro/Ala at P2; such peptides, representing a little amount of the HLA-B*51 peptidome and consisting of Gly, Ser or Val in P2, increase when ERAP1 is silenced [68]. Moreover, the HLA-B*51 peptidome comprises also 8-, 10- and 11-mers, although representing ligands with lower affinity [69]. It is therefore reasonable to speculate that such variation in the peptide repertoire could favor the formation of altered peptide/HLA-B*51 complexes, which could induce CD8^+^ T cell activation [70]. Comparing the length of non-Pro/Ala2 peptides with Pro2 and Ala2 sub-peptidomes, Pro2 peptides are longer than the other two groups (Ala2 and non-Pro/Ala2), whereas the non-Pro/Ala2 ones are frequently 8-mers, with a low percentage of 9- or 10-mers. This could be explained considering that ERAP1 does not preferentially cut substrates carrying Pro in P2 [67,68]. As for HLA-B*27, there is a strong epistatic relationship between HLA-B*51 and ERAP1, since the association of ERAP1 with the disease stands only in HLA-B*51-positive patients with BD [13]. No evidence supports a role for ERAP2 in the pathogenesis of BD and this is consistent with the absence of basic residues at P1 among HLA-B*51 ligands [18]. This could be related, at least in part, to the different effects of these aminopeptidases on the peptidome of HLA-B*51:01. In fact, recent data based on the deletion of ERAP1, ERAP2 or both through CRISPR/Cas9 revealed that, beside a large amount of the HLA-B*51:01 ligandome that is independent from ERAP1 or ERAP2 trimming, the two ERAPs exert distinct but complementary and partially redundant effects [71].

The Ala2 and Pro2 sub-peptidomes differ also for the P1 residues and the balance between these two large sub-peptidomes depends on the activity of ERAP1; in fact, the former has a prevalence of Asp in P1, while the latter has aliphatic/aromatic residues in P1. Notably, this difference is influenced by the activity of ERAP1: aliphatic/aromatic residues (except Val) are highly susceptible to the trimming of ERAP1, whereas X-Pro bond are not cut and are good anchor residues for HLA-B*51; instead, Ala is a good substrate for ERAP1 but residues such as Asp in P1 are resistant [67,72]. The absence of ERAP1 also affects the length of the peptides with an increase in peptides of 10- and 11-mers [68]. Furthermore, the risk variant of ERAP1 (Hap10) could favor longer epitopes, an increase in the Ala2 sub-peptidome and an overall lower affinity for HLA-B*51 ligands [73]. Interestingly, the major risk haplotype of ERAP1 (Hap10) in BD, implying a low active ERAP1 variant, turns out to be protective in AS [11,13]. Seemingly, the effect of ERAP1 is allele-dependent and for HLA-B*51 the low activity of ERAP1 could preserve suboptimal peptides inducing harmful CD8^+^ T cell responses. However, the effect of ERAP1 on the expression of HLA-B*51 needs further investigations, since the knockdown of ERAP1 have different outcomes in different cell types [68].

Despite several studies aimed at a deeper understanding of the BD basis, the contribution of HLA-B*51 and ERAP1 to the disease risk could be consistent with the involvement of a dysregulated CD8^+^ T response or with an aberrant NK cell activation likely caused by altered interactions with inhibitory leucocyte immunoglobulin-like receptors (LILRs) or killer immunoglobulin-like receptors (KIRs) [65]. It cannot be excluded that such dysregulated responses could be raised by unconventional peptides bound to HLA-B*51. Disclosing the extent of such repertoire can be helpful to clarify the contribution of HLA-B*51 as antigen presenting molecule in predisposing to BD.

## 4. HLA-A*29 Suboptimal Peptidome: Contribution to the Pathogenesis of Birdshot Chorioretinopathy

The term Birdshot Chorioretinopathy (BSCR) was introduced in 1980 by Ryan and Maumenee to describe a bilateral chronic intraocular inflammation or posterior uveitis that preferentially affects middle-aged Caucasians [74,75]. Likewise, AS and BD, the BSCR is strongly associated with an HLA class I allele, HLA-A*29, and virtually all patients are positive for this allele [76,77]. The association between BSCR and HLA-A*29 supports a pathogenetic role for this allele in its antigen presentation function, but the exact contribution remains elusive. In line with this hypothesis, it has been proposed that HLA-A*29 could induce autoreactive T cell responses towards retinal targets by molecular mimicry with microbial epitopes [78]. Additionally, GWAS identified functional genetic variants of ERAP1 and ERAP2 as risk factors for BSCR [14] that, together with HLA-A*29 association, might affect antigen processing and/or presentation, leading to an altered immune response [14,79].

To date, about twenty HLA-A*29 subtypes have been identified with HLA-A*29:01 and HLA-A*29:02 more represented in the Asian and Caucasian population, respectively. Interestingly, similarly to HLA-B*27:05 and HLA-B*27:09, these two HLA-A*29 alleles differ for a single substitution at the residue 102, but are both associated with the disease and share the spectrum of bound peptides [80,81]. Notably, it has been ascribed to HLA-A*29 the “supremacy” in the binding of overlapping viral epitopes at the expense of other HLA class I molecules [82]. Moreover, the HLA-A*29 has also been predicted to bind viral peptides originally described as restricted for other HLA class I molecules [83]. These observations suggest a beneficial role of HLA-A*29 during some viral infections; however, conversely to HLA-B*27, the expression of the HLA-A*29 molecules results in a rapid progression to AIDS in HIV-positive individuals [84]. Once again, linking the role of HLA-A*29 in the viral defense to its association to BSCR, giving special attention to CD8^+^ T cell responses, can be the right approach to the molecular dissection of the “MHC-I-opathies”.

In order to be good HLA-A*29:02 binders, peptides must possess at least a Tyr as P9; in fact, the Tyr alone is sufficient to ensure the attachment of the peptides to the HLA-A*29 binding groove [18,85]. Such weak restriction could expand the hypothetical pathogenetic peptidome, probably derived from the eye microenvironment. Such peptides could be strictly dependent on ERAP2 trimming, since the risk associated to BSCR implies the presence of a highly active ERAP2 associated with the low active variants of ERAP1 (Hap10) [14,79]. The risk peptidome derived from this context is expected to be enriched in longer suboptimal peptides with lower affinity to HLA-A*29:02. Indeed, studies conducted in the absence of ERAP2 combined with high performing ERAP1 variants showed an increase of nonamers possessing higher affinity to HLA-A*29:02 [18,86]. In agreement, data obtained in a context of highly active allelic variants of both ERAP1 and ERAP2 detected an increment of peptides longer than 9 aa with P1 residues less susceptible to ERAP2 and more hydrophobic [87]. These observations, added to the global non-restrictive HLA-A*29:02 binding motif, argue for the implication of a “mis-immunopeptidome” that, in the eye microenvironment, could sustain an autoreactive CD8^+^ T cell response.

Indeed, a role for T cells in the eye damage is sustained by the finding of infiltrated CD8^+^ and CD4^+^ T cells in the vitreous fluid samples of BSCR subjects [88]. Moreover, it has been demonstrated that retinal and choroidal lysates could induce the response of such intraocular T cells, but the relevant autoantigens remain to be identified [88].

## 5. HLA-C*06:02 and Psoriasis: The Impact of Autoreactive CD8^+^ T Cells

Psoriasis is a complex immune-mediated disorder in which keratinocyte hyperplasia and chronic inflammation of the skin, manifesting with plaques and papules, are considered as primarily driven by T-cell activation [89,90]. Although GWAS have identified multiple risk genetic variants, which affect pro-inflammatory pathways, antigen processing and presentation, cell signaling, transcriptional regulation together with epidermal function and differentiation, the causes of the disease are not yet clear [91].

The predominant risk factor to develop the disease is represented by HLA-C*06:02, found in more than 60% of psoriatic patients. Its role in the pathogenesis is still unclear. It could be involved in an aberrant functioning of the innate immune system through an interaction of HLA-C*06:02 with KIR2DL1, an inhibitory receptor expressed on NK and T natural killer cells, leading to an abnormal activity of innate lymphoid cells [92]. In alternative, but not mutually exclusive, its role in the disease could be related to the antigen presentation to CD8^+^ T cells, as supported by recent studies [93,94].

HLA-C*06:02 generally prefers nonamers and longer peptides are rarely presented by this molecule. The peptides are characterized by an Arg in P2 and P7, whereas small hydrophobic residues such as Val, Leu and Ile are preferred at the C-terminus. Mobbs et al. characterized the structure of HLA-C*06:02 highlighting a deep peptide-binding groove that accommodates the electronegative B and E pockets, which explains the preference for an Arg in P2 and P7 [95].

Noteworthy, HLA-B*27, which is among the HLA class I alleles predisposing to Psoriasis, presents an immunopeptidome partly overlapping with that of HLA-C*06:02 characterized by shared anchor residues at P2 and P9 [96]. Other risk factors in Psoriasis are allelic variants of ERAP1, such as rs27524 (noncoding) and rs30187 (K528) (in epistasis with HLA-C*06:02), included in the Hap2 risk haplotype [15]. Nevertheless, the association with ERAP2 and the effects of these two aminopeptidases on the HLA-C*06:02 peptidome need to be further elucidated [18]. Since psoriatic lesions are enriched in activated CD8^+^ T cells displaying strong oligoclonality [97,98], in recent years, there has been an increasing interest in finding potential CD8^+^ T cell autoantigens that trigger the autoimmune reactivity. At least three putative autoantigens implicated in Psoriasis have been identified so far: cathelicin LL37 [93], disintegrin-like and metalloprotease domain containing thrombospondin type 1 motif-like 5 (ADAMTSL5) [99] and keratin 17, whose involvement is mainly based on the homology to streptococcal M-proteins [94], since streptococcal infection is considered a major environmental trigger of the disease. Interestingly, all these proteins are overexpressed in psoriatic skin [100,101]. The LL37 is an antimicrobial peptide that, in complex with self-DNA, stimulates plasmacytoid dendritic cells (pDC) to produce type I interferons (IFNs) which, in turn, mature and activate myeloid DC (mDC), thus promoting autoimmunity by expanding autoreactive T cells [102]. ADAMTSL5, instead, is a protein expressed mainly by melanocytes and involved in the modulation of microfibrils. An elegant work by Arawaka and co-workers demonstrated for the first time that ADAMTSL5 is a melanocyte autoantigen in Psoriasis [99]. By using peptide library screening, they identified a self-peptide derived from ADAMTSL5 as the autoantigen recognized in the context of HLA-C*06:02 by a Vα3S1/Vβ13S1 T cell receptor (TCR) expressed by an epidermal CD8^+^ T cell clone derived from a HLA-C*06:02-positive patient and directed against melanocytes. Notably, only blood lymphocytes of psoriatic patients but not healthy subjects responded to stimulation with ADAMTSL5 by secreting IFN-γ and IL-17A [99,103].

The identification of specific autoantigens relaunches the main role of HLA-C*06:02 in activating autoreactive CD8^+^ T cells as a concrete mechanism implicated in Ps pathogenesis. Although such autoantigens possess a proper consensus sequence, the possibility to find other candidate epitopes not possessing the canonical HLA-C*06:02 binding motif cannot be excluded.

## 6. Conclusions

**Figure 1:**
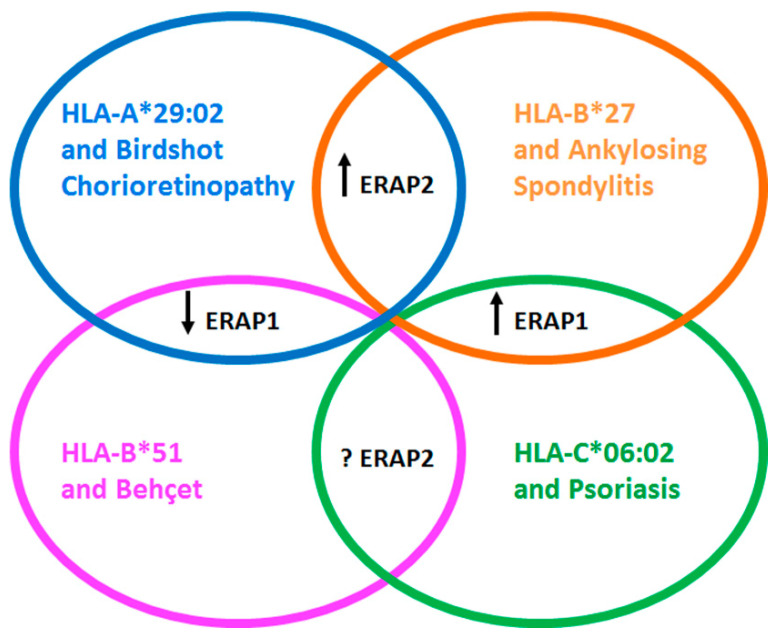
The crosstalk between different variants of ERAP1 and ERAP2 and the HLA class I molecules: contribution to the autoinflammatory/autoimmune diseases. Ankylosing Spondylitis (AS), Psoriasis (Ps), Birdshot Chorioretinopathy (BSCR) and Behçet’s disease (BD) share the association with variants of ERAP1 and ERAP2, beside the strong link with a specific HLA class I molecule, that is HLA-B*27, HLA-C*06:02, HLA-A*29:02 and HLA-B*51, respectively. Intriguingly, the contribution of ERAP1 is related both to the high or low enzymatic activity variant, partially due to the ligandome of the HLA class I involved in the disease; in fact, the former represents a risk for AS and Ps, while the latter is predisposing for BSCR and BD. The role of ERAP2 is still under investigation with no evidence of a compelling involvement in Ps and BD, while its presence seems to predispose to AS and BSCR, although combined with high or low variants of ERAP1, respectively. The arrows pointing upwards indicate allelic variants with high enzymatic activity; the arrow pointing downwards indicates allelic variants with low enzymatic activity; the question mark denotes no evidence of disease association as for BD or absence of compelling data defining the risk alleles as in Ps.

The main role of ERAP1 is to refine the peptides bound to HLA class I molecules and presented to specific CD8^+^ T cells; although ERAP2 is part of this processing, its effects only partially overlap with those of ERAP1. Of note, ERAP2, differently from ERAP1, is not in epistasis with the disease-associated HLA class I molecules. However, considering its absence in about 25% of the population, it is reasonable to speculate about its involvement in additional pathogenetic mechanisms. Interestingly, the pathogenesis of autoimmune/autoinflammatory disorders, such as AS, BD, BSCR and Ps, seems to arise, at least in part, by the epistatic interaction between ERAP1 and a HLA class I molecules (HLA-B*27, HLA-B*51, HLA-A*29:02 and HLA-C*06:02, respectively). This could be related to the specific peptidome of each HLA class I types. In fact, HLA-B*27 and HLA-C*06:02, which partially share their binding motif, associate with the same highly active ERAP1 haplotypes. On the opposite, low active forms of ERAP1 are involved in the onset of BSCR and BD thus subdividing these “MHC-I-opathies” in two categories where, in any case, ERAP1 plays a pivotal role in the occurrence of the “mis-immunopeptidome” with a pathogenetic relevance (Figure 1).

Interestingly enough, the presence of ERAP2 seems to strengthen the pathogenetic effects of ERAP1, at least in the case of AS and BSCR, while in BD and Ps its contribution is not relevant or not yet clear (Figure 1). Such cross-talk could favor the switching of an optimal ligandome towards a “mis-immunopeptidome” that, in turn, could influence the immune response, both adaptive (CD8^+^ T cells) and innate (NK cells), but might also imbalance the regulatory counterpart of the immune system.

It would certainly be desirable to find specific pathogenetic epitopes towards which to guide a possible immune therapy and, at the same time, it could be useful combining it with specific drugs able to inhibit, such as DG013A does [104], or enhance ERAP1 and ERAP2 activity depending on the variants associated with the specific disease.

In conclusion, the integration of genetic data with functional genomics, immunology, drug design and clinical practice will ultimately lead to fundamental advances in understanding critical molecular pathways and improving disease therapy.

## Abbreviations

HLA	Human Leucocyte Antigen
ERAP	Endoplasmic Reticulum Aminopeptidase
MHC	Major Histocompatibility Complex
AS	Ankylosing Spondylitis
SpA	Spondyloarthropathies
BD	Behçet’s Disease
Ps	Psoriasis
BSCR	Birdshot Chorioretinopathy
PsA	Psoriatic Arthritis
ReA	Reactive Arthritis
IBD	Inflammatory Bowel Disease
GWAS	Genome-Wide Association Studies
NK	Natural Killer
SNP	Single Nucleotide Polymorphisms
CTL	CD8^+^ T Lymphocyte
HIV	Human Immunodeficiency Virus
AIDS	Acquired Immuno-Deficiency Syndrome
HCV	Hepatitis C Virus
EBV	Epstein-Barr Virus
CMV	Cytomegalovirus
ER	Endoplasmic Reticulum
FHC	Free Heavy Chain
TAP	Transporter Associated with antigen Processing
CRISPR	Clustered Regularly Interspaced Short Palindromic Repeats
LILR	Leucocyte Immunoglobulin-Like Receptors
KIR	Killer Immunoglobulin-like Receptors
ADAMTSL5	A Disintegrin-like and Metalloprotease domain containing Thrombospondin Type 1 motif-like 5
DC	Dendritic Cell
IFN	Interferon
IL	Interleukin
